# First serine protease inhibitor isolated from *Rhinella schneideri* poison

**DOI:** 10.1186/s40409-015-0029-4

**Published:** 2015-08-13

**Authors:** Priscila Y T Shibao, Fernando A P Anjolette, Norberto P. Lopes, Eliane C. Arantes

**Affiliations:** Department of Physics and Chemistry, School of Pharmaceutical Sciences of Ribeirão Preto, University of São Paulo (USP), Avenida do Café, s/n, 14.040-903 Ribeirão Preto, SP Brazil

**Keywords:** Bufadienolide, Rhinella schneideri, Serine protease inhibitor

## Abstract

**Background:**

Toad secretions are a source of molecules with potential biotechnological application on a wide spectrum of diseases. Toads from the *Rhinella* family have two kinds of poisonous glands, namely granular and mucous glands. *Rhinella schneideri* toads produce granular secretions that comprise a great number of molecules, including serine proteases inhibitors. Serine proteases, such as trypsin, chymotrypsin and elastase, are enzymes that have a serine amino acid into its catalytic site and can be found in a large number of vertebrate species and pathogenic microorganisms. Therefore, the present work aims to purify a serine protease inhibitor from *Rhinella schneideri* granular secretions.

**Findings:**

This study presents the protocol used to purify a serine protease inhibitor from the *Rhinella schneideri* poison. The granular secretion was submitted to dialysis in order to separate the low molecular weight compounds, which were submitted to a reversed phase-fast protein liquid chromatography fractionation step in a C2C18 column. The major fractions were tested over trypsin, chymotrypsin and elastase through colorimetric assay. The inhibition tests were performed with the enzyme in absence (positive control) and presence of fractions, denatured enzyme (negative control) and the respective chromogenic substrate. Rs20 was the compound with the major inhibitory activity over chymotrypsin, inducing a delay in the formation of the chromogenic enzymatic product. The structure characterization of Rs20 was performed by high resolution electronspray ionization-mass spectrometry (HRESI-MS) and gas chromatography coupled with mass spectrometry (GC-MS). HRESI showed an intense signal suggesting the presence of bufadienolide with less than 10 ppm error. In addition, it was observed a low intense signal at *m/z* 399 that could be lithocholic acid, a biosynthetic precursor of bufadienolide. Finally, GC-MS analysis applying NIST library identification reinforced this hypothesis.

**Conclusions:**

The current study have isolated and partially characterized the function and structure of the first bufadienolide with inhibitory action over chymotrypsin.

**Electronic supplementary material:**

The online version of this article (doi:10.1186/s40409-015-0029-4) contains supplementary material, which is available to authorized users.

## Background

*Rhinella schneideri* toads, that are widespread throughout the South American territory, have two types of glands: granular and mucous. Mucous glands can be found along the entire animal body and their secretion aid toad survival in inhospitable habitats, since they assist in skin hydration and gas exchange [1].

The secretion of granular glands, or parotoid glands, is responsible for the toad passive defense against predators [2]. This poison is composed of biogenic amines, steroids, alkaloids, peptides and proteins [3]. Whereas steroids are responsible for accelerating the heart rate of affected animals, inducing apoptosis and hallucinogenic effects, the peptides and proteins are believed to improve toad defense against microorganisms [4]. Most studies focus on this kind of poison due to its composition and diversity of effects induced by their components [5].

Serine proteases enzymes present an amino acid serine on its catalytic site. The serine interacts with a carbonyl group from the substrate, facilitating an acyl group transfer that results in a peptide cleavage [6]. Such enzymes are related to several functions that are intrinsic to homeostasis [7–10]. It is expected that animals that lived in such an inhospitable habitat have created their own arsenal of serine protease inhibitors along the course of time, which is named evolutionary arms race [11]. Previous studies with Anurans have demonstrated the presence of serine protease inhibitor in their secretions [12–15].

## Findings

### Poison

Adult specimens (n = 5) of *Rhinella schneideri* from the animal facility of the University of São Paulo in Ribeirão Preto, were used for poison extraction. It was performed by mechanical pressure on the parotoid glands. The secretion (pool of five extractions) was dried and stored at–20 °C. A poison sample can be found at the venom bank of the Center for the Study of Venoms and Venomous Animals (CEVAP/UNESP). The poison suspension (500 mg in 20 mL of water) was dialyzed on a 6–8 kDa pore membrane against deionized water. The dialysis water containing the molecules that permeate the membrane (molecular weight < 8 kDa) was frozen, lyophilized and stored at–20 °C.

### Purification of a serine protease inhibitor

The sample (3 mg) obtained from dialysis water was dispersed in 40 μL of acetonitrile and then 0.1 % trifluoracetic acid (TFA) solution (360 μL) and water (600 μL) were added to the solution completing 1 mL. The solution was submitted to reversed-phase fast protein liquid chromatography (RP-FPLC) using a C2C18 column (μRPC C2/C18 ST 4.6/100, Amersham Biosciences, Sweden) equilibrated with 0.1 % TFA (solution A), at flow rate of 1 mL/min. Fractions elution was monitored at 280 nm in ÄktaBasic UPC system (GE, Sweden).

### High resolution electronspray ionization-mass spectrometry (HRESI-MS)

The sample called Rs20 was analyzed in a high resolution eletronspray mass spectrometer (HRESI-MS) (Bruker Daltonic, USA) and was direct infused in the ESI source though a syringe pump (Kd Scientific, USA). Nitrogen was used as dry gas at 180 °C (under 4 L/min flow) and as nebulization gas (pressure of 0.4 Bar). The capillary voltage was set up to 3500 volts. Sodium trifluoracetate was applied as internal calibration before the data.

### Gas chromatography coupled with mass spectrometry analyzer (GC/MS)

Rs20 was also analyzed in GC/MS equipment (Shimadzu QP2010, Japan). One microliter of sample solutions was injected at 220 °C in a HP-5MS column (30 m × 0.25 mm × 0.25 μm; Agilent Technologies). The analysis was performed with two minute sample time in splitless injection mode, column flow of 1.28 mL/min, linear velocity of 44.1 cm/sec, and scan between *m/z* 40.00 and *m/z* 500.00. The oven temperature started with 220 °C for six minutes, increasing to 8 °C min^−1^ until 280 °C, this temperature was maintained for 20 minutes, then increased to 15 °C min^−1^ until 310 °C and then kept for 40 minutes. Lithocholic acid structure was proposed by comparison with NIST library.

### Serine protease inhibitory assay

The major fractions Rs1, Rs4, Rs7, Rs9, Rs10, Rs15, Rs16, Rs17, Rs20 and Rs22 from the RP-FPLC fractionation were submitted to inhibitory activity assay with the three serine proteases, porcine trypsin, chymotrypsin and porcine elastase. Inhibitory activity assay was performed by mixing the fractions (1 mg/mL, 100 μL) with each enzyme (1 mg/mL, 50 μL) and PBS buffer (50 μL) and incubating for 15 minutes at room temperature. Enzyme activity was determined by adding the correspondent chromogenic substrate (1 mg/mL, 100 μL). The substrates used were N-Tosylglycyl-L-prolyl-L-lysine 4-nitroanilide acetate salt, N-Succinyl-Ala-Ala-Pro-Phe-4-nitroanilide and N-Succinyl-Ala-Ala-Ala-p-nitroanilide for porcine trypsin, chymotrypsin and elastase, respectively. The formation of p-nitroanilide was monitored at 405 nm, every two minutes, for 60 minutes. Positive (enzyme, buffer and corresponding substrate) and negative (fraction, denatured enzyme, buffer and corresponding substrate) controls, and Rs20 enzymatic action (fraction, buffer and substrate) were performed. The poison enzymatic inhibitory assay was also performed by mixing poison solution (10 mg/mL, 100 μL) with the enzymes and PBS buffer, as described for the assays with fractions. Assays were performed only once in triplicate.

### Statistical analysis

The results were expressed as the mean ± SEM. Statistical comparisons among the groups were carried out using ANOVA followed by the student’s *t*-test. Data were statistically significant when p < 0.05. All data analyses were done using Prism™ v.5 (GraphPad Inc., USA).

## Conclusions

### Purification of serine proteases inhibitor

**Fig. 1:**
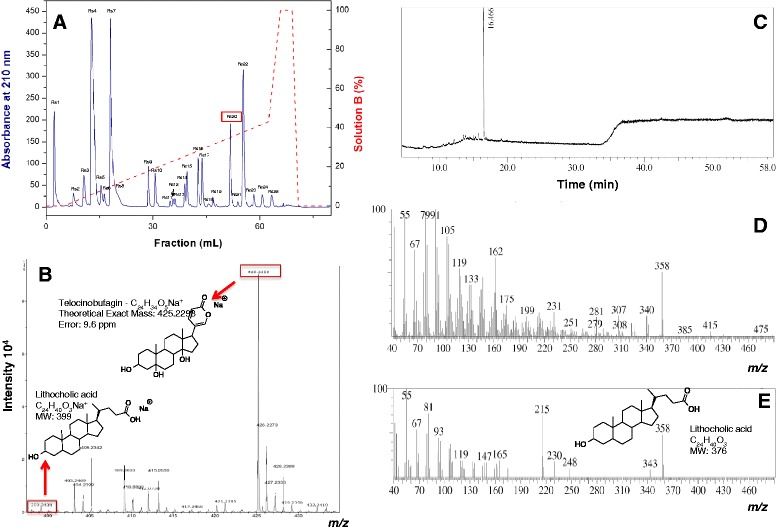
RP-FPLC profile and structural characterization of Rs20 by HRESI-MS and GC/MS. **a**. RP-FPLC on a C2C18 column previously equilibrated with solution A (TFA 0.1 %, V/V). The fractions were eluted with a concentration gradient of solution B (acetonitrile 80 % in TFA 0.1 %, V/V) until it reaches 100 % of solution B (dashed line). Flow rate: 1 mL/minute. **b**. ESI-microTOF mass spectrum of Rs20. The spectrum indicates the ion masses obtained through positive mode. **c**. Gas chromatography profile. The spectrum is shown in total ion chromatogram (TIC) × time (minute). **d**. GC/MS mass spectrum of Rs20. **e**. GC/MS mass spectrum of lithocholic acid

The purification by RP-FPLC resulted in 25 fractions (Fig. 1a), Rs1 to Rs25. Each fraction was frozen and lyophilized.

### Mass spectrometry analysis

The fraction Rs20 showed the highest serine protease inhibition over chymotrypsin and was submitted to ESI-TOF spectrometer. The HRESI-MS (Fig. 1b) shows a relevant ion with *m/z* of 425.2258. This ion matches with telocinobufagin molecular weight with a sodium adduct [16]. The analysis was performed in high-resolution equipment and the error (9.6 ppm) was determined. This result suggests telocinobufagin as a possible inhibitor. Another signal was observed in ESI analysis, but at low intensity (*m/z* 399) which can be related to the lithocholic acid (Fig. 1b inset), a biosynthetic precursor of telocinobufagin. At GC-MS analysis (Fig. 1c, d and e), telocinobufagin was not observed probably by the low volatilization of its structure. In the other hand, one signal was observed at 16.4 minutes (Fig. 1c) which can be related to lithocholic acid. As expected, the molecular ion was not observed, only the water elimination fragment (Fig. 1d). Comparison with the data in NIST-08 spectrum library showed similar behavior (80 % of similarity), suggesting the presence of the lithocholic acid (Fig. 1e).

### Inhibitory assay

**Fig. 2:**
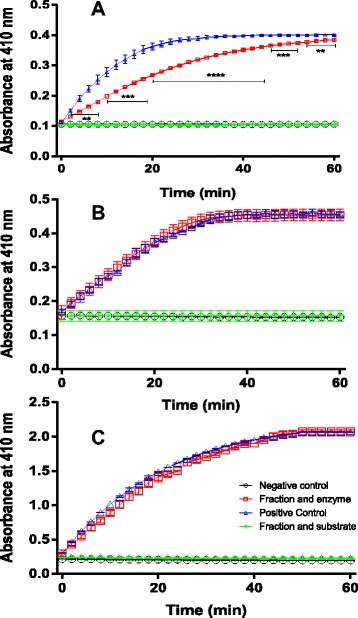
Inhibition assays. **a**. Enzyme inhibition assay of Rs20 over trypsin. **b**. Enzyme inhibition assay of Rs20 over chymotrypsin. **c**. Enzyme inhibition assay of Rs20 over elastase. In the enzyme inhibition assays,  indicates positive control (enzyme, PBS buffer and substrate);  indicates assay performed in the presence of Rs20 (enzyme, Rs20, PBS buffer and substrate);  indicates negative control (denatured enzyme, PBS buffer and substrate); and  indicates the enzymatic action of Rs20 (Rs20, buffer and substrate). The points represent the means ± SEM of one experiment performed in triplicate. ** p < 0.01; *** p < 0.001 and **** p < 0.0001 compared to the positive control

Major fractions obtained from the RP-FPLC were tested over the activity of three serine proteases (data not shown). Rs20 showed significant inhibitory activity (p < 0.0001) over chymotrypsin (Fig. 2a). Conversely, it did not show inhibition over elastase (Fig. 2b) or trypsin (Fig. 2c). The poison was able to inhibit both trypsin and chymotrypsin, with different potencies (Additional file 1). Assays were performed only once in triplicate.

In this study, we isolated a poison component (Rs20) with inhibitory activity over chymotrypsin. The poison should have more than one serine protease inhibitor, since it was able to delay the p-nitroanilide formation by trypsin and chymotrypsin while Rs20 has shown a significant inhibition only over chymotrypsin. Protease inhibitors can be of protein or non-protein nature. The protein inhibitors include baserpins, Kunitz inhibitors, BSTI and others [13–19].

Triterpenes, phenolic compounds and alkaloids are among non-protein molecules that may be found in amphibian secretions, including toads from *Rhinella* family. Daly et al. [20] listed more than 800 hundred alkaloids present in amphibian secretions and skin. Their occurrence may be explained by a bioaccumulation process originated in the food chain: plants are able to synthesize these kind of compounds through secondary metabolism, they are eaten by insects that are preyed by amphibians [21].

The purification strategy started with the poison dialysis in a 6–8 kDa membrane, followed by RP-FPLC of the sample containing low molecular weight compounds (Fig. 1a). This protocol was able to successfully fractionate the poison in 25 fractions (Rs1 to Rs22) with a high-resolution profile.

Rs20 showed the greatest inhibitory activity over chymotrypsin (Fig. 2b) and was chosen for structural characterization through electrospray ionization and gas chromatography coupled with a mass spectrometry analyzer. The HRESI-MS spectrum (Fig. 1b) suggested that Rs20 is a bufadienolide, probably the telocinobufagin structure (Fig. 1b inset) [16]. The major ion *m/z* 425.2258 corresponds to the telocinobufagin (molecular formula: C_24_H_34_O_5_) with a sodium adduct [M + Na]^+^, showing an error of 9.6 ppm [22]. Another minor ion was observed in HRESI-MS analysis suggesting the presence of the lithocholic acid (Fig. 1b inset). Finally, GC-MS analysis (Fig. 1c, d and e) supported by NIST-08 spectrum library suggested the presence of the lithocholic acid a biosynthetic precursor of telocinobufagin.

Several biological actions have been reported for telocinobufagin, such as antiproliferative activity towards human cancer cells, antileishmanial activity and even immunomodulatory activity [23–25]. This work showed that this bufadienolide is also able to inhibit selectively chymotrypsin (Fig. 2a), which is a new functional feature of it. It would be expected that Rs20 would be able to inhibit trypsin activity, once they share almost the same structure, with difference of two amino acids at their catalytic site. Trypsin presents arginine at the 117th position and valine at the 118th position, while chymotrypsin presents a phenylalanine at 114th position and serine at 115th position [26, 27]. Conversely, it did not inhibit trypsin (Fig. 2b) or elastase (Fig. 2c). Probably, the slight changes at the catalytic site of chymotrypsin increases the affinity between the bufadienolide and the enzyme.

In conclusion, this study have isolated and partially characterized the function and structure of the first bufadienolide with inhibitory action over chymotrypsin, isolated from *Rhinella schneideri* poison.

## Additional file

Additional file 1:
**Poison inhibitory assays.** (DOCX 725 kb)
