# Supplementary material for: First serine protease inhibitor isolated from Rhinella schneideri poison
Source: J Venom Anim Toxins Incl Trop Dis. 2015 Aug 13;21:30. doi: 10.1186/s40409-015-0029-4 (PMC4535736; doi:10.1186/s40409-015-0029-4)
Supplement: Additional file 1: — Poison inhibitory assays. (DOCX 725 kb) [file 40409_2015_29_MOESM1_ESM.docx]

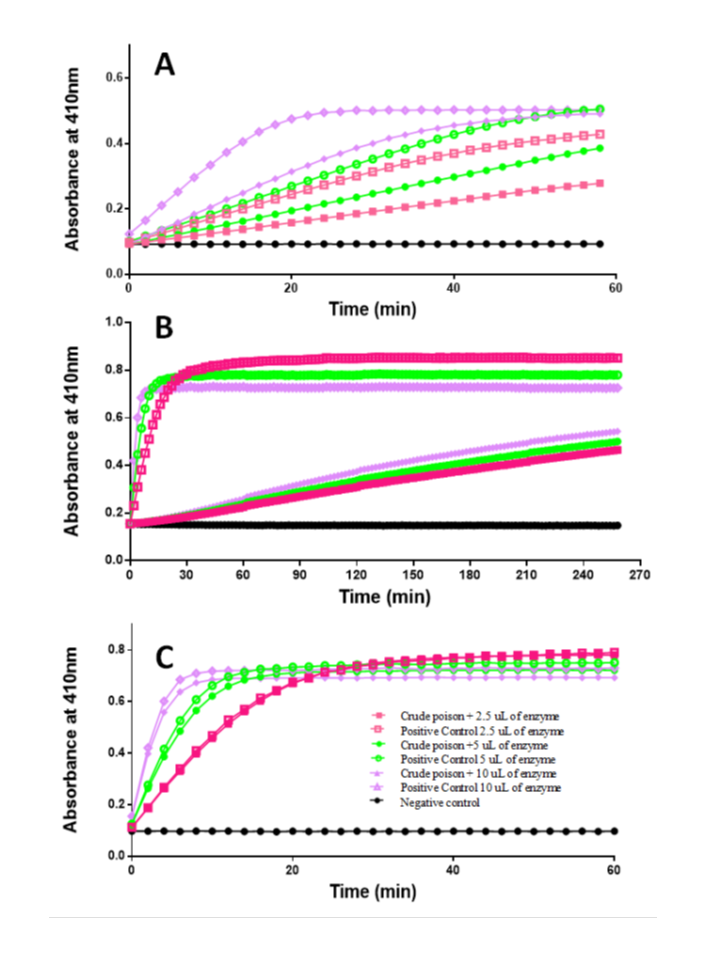


**Additional file 1. Poison inhibitory assays. (A)** Enzyme inhibition assay of poison over trypsin. **(B)** Enzyme inhibition assay of poison over chymotrypsin. **(C)** Enzyme inhibition assay of poison over elastase. In the enzyme inhibition assays,
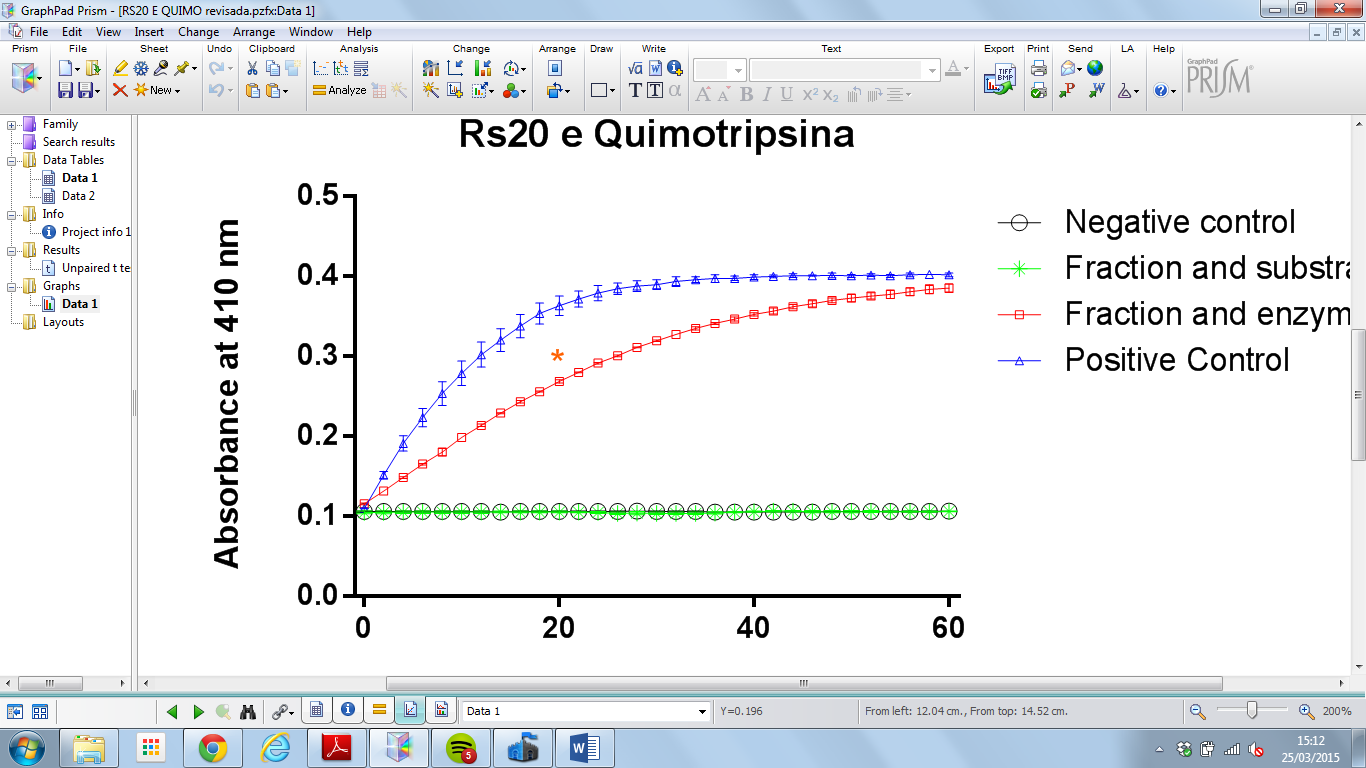
indicates positive control (enzyme, PBS buffer and substrate) for 2.5 μL of enzyme solution (1 mg/mL);
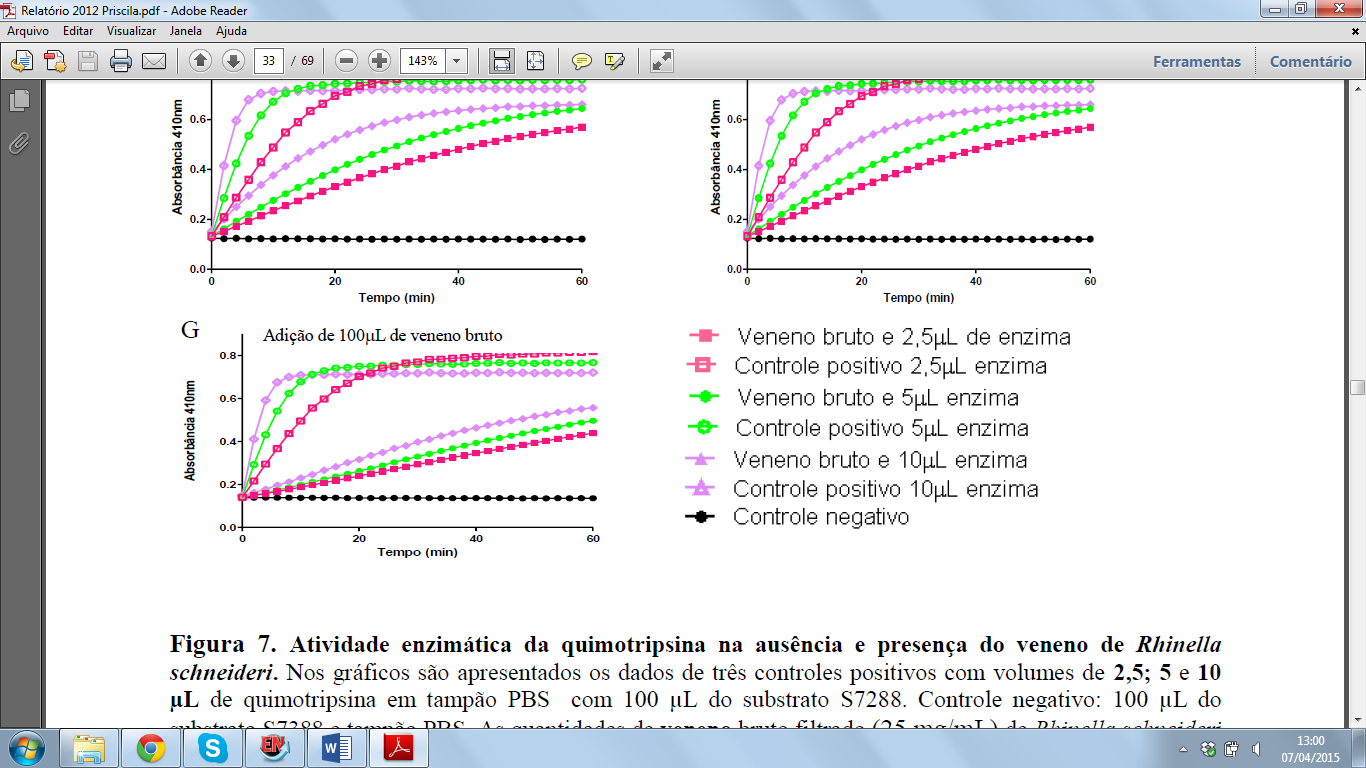
indicates assay performed in the presence of poison and 2.5 μL of enzyme solution (1 mg/mL) (enzyme, poison, PBS buffer and substrate);
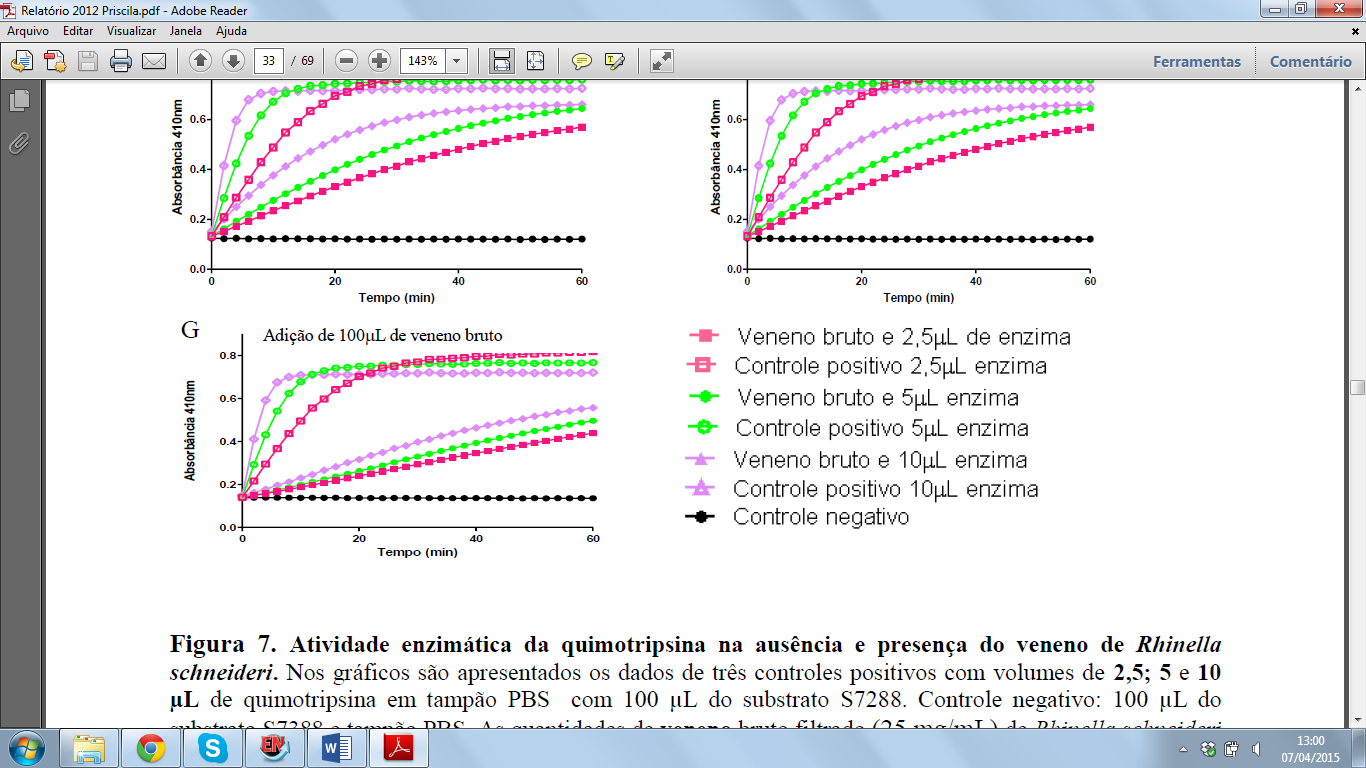
 indicates positive control for 5.0 μL of enzyme solution;
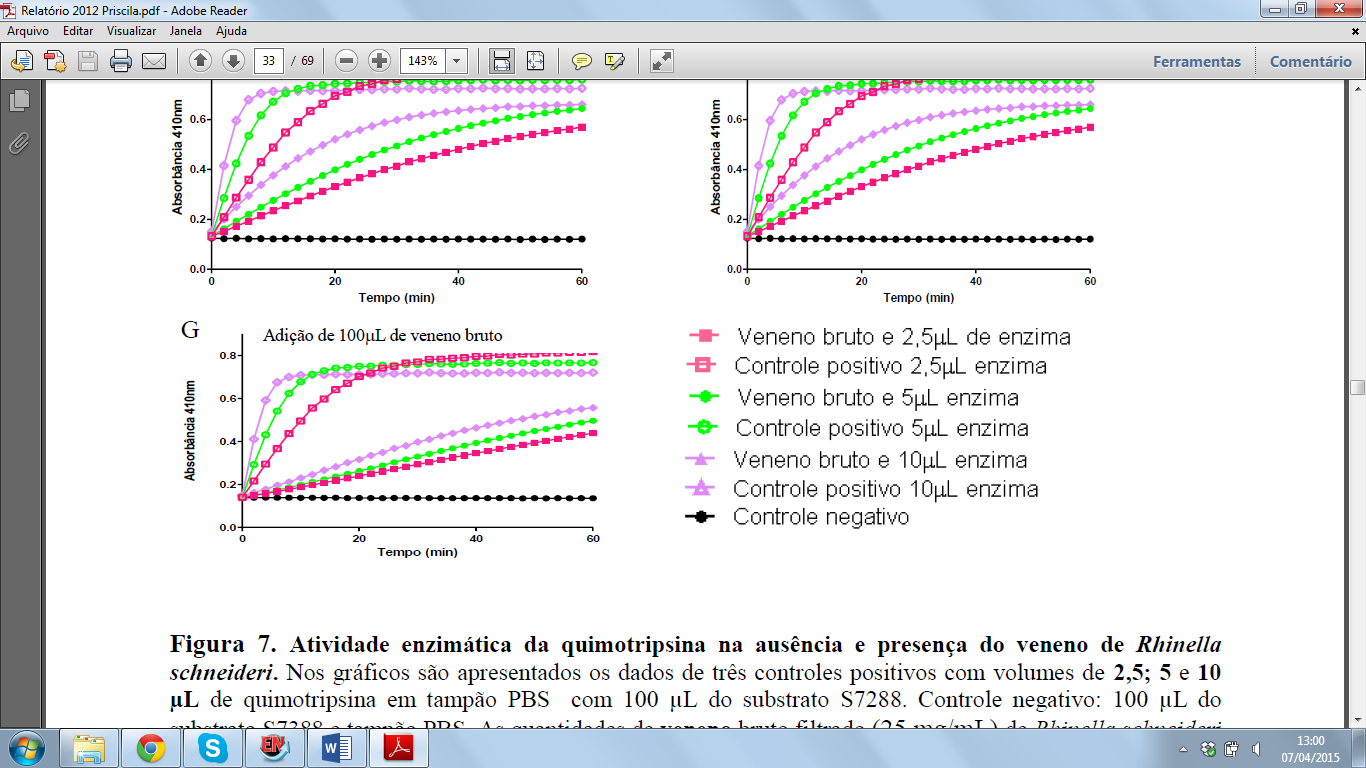
 indicates assay performed in the presence of whole venom and 5.0 μL of enzyme solution;
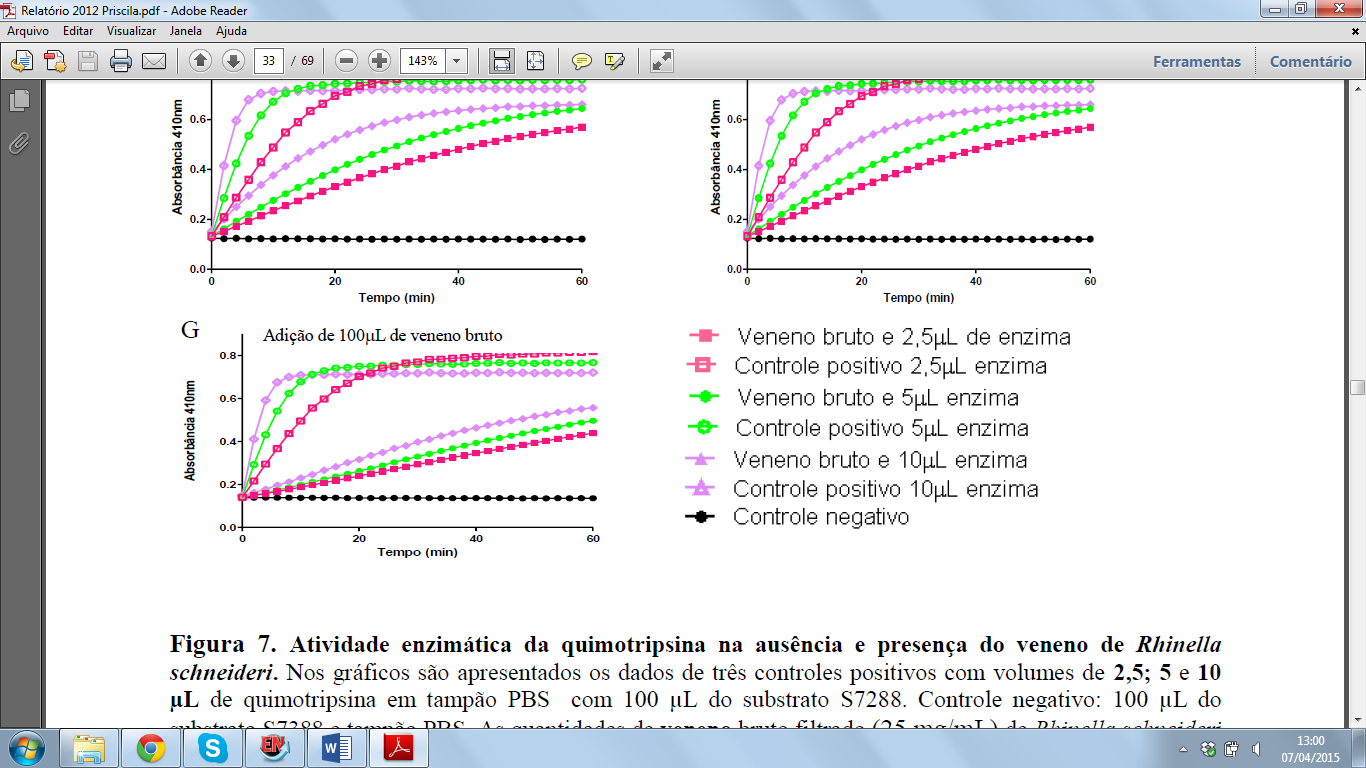
 indicates positive control for 10.0 μL of enzyme solution;
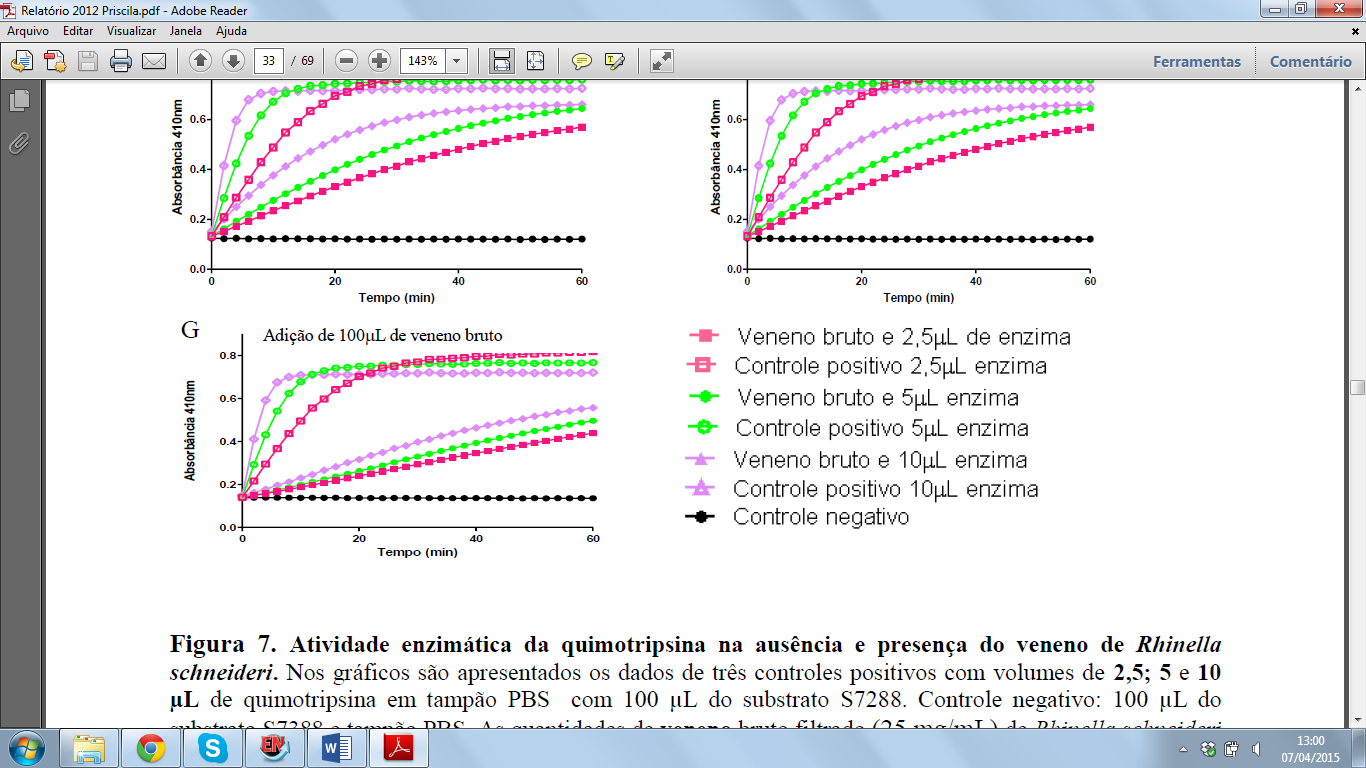
indicates assay performed in the presence of whole venom and 10.0 μL of enzyme;
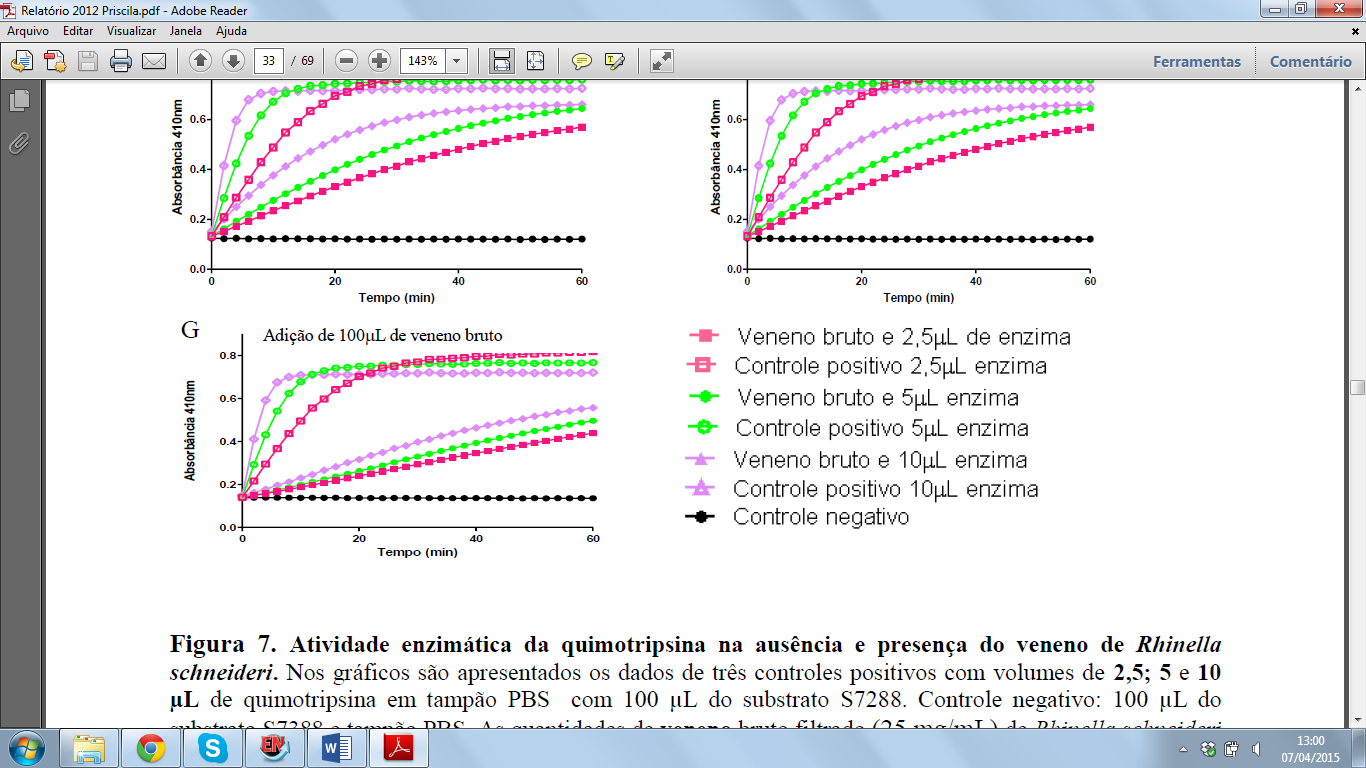
indicates negative control (denatured enzyme, PBS buffer and substrate). Trypsin and elastase assays were performed once in triplicate. Chymotrypsin assay was performed twice in triplicate.
